# When Delirium Is Not Delirium: A Case of Late-Life Mania Initially Attributed to Medication Effects

**DOI:** 10.1192/bjo.2026.11881

**Published:** 2026-06-30

**Authors:** Shalina Ramsewak, Shruti Lodhi

**Affiliations:** Surrey and Borders Partnership NHS Trust, Surrey, United Kingdom

## Abstract

**Aims:**

Acute behavioural disturbance in older adults frequently presents diagnostic challenges, particularly when symptoms arise in the context of polypharmacy, physical illness, and sleep disruption. Delirium is often the initial working diagnosis in such presentations; however, evolving clinical features may indicate an underlying primary psychiatric disorder. Distinguishing between delirium and late-onset affective illness is especially complex in older adults with pre-existing psychiatric vulnerability and medication exposure.

**Methods:**

This case describes a 67-year-old woman with a background of recurrent depressive disorder, anxiety, and obsessive–compulsive disorder who presented with acute confusion, severe insomnia, behavioural disorganisation, and pressured speech following recent changes to her analgesic regimen, including the initiation of tramadol.

The initial presentation was characterised by fluctuating cognition, psychomotor agitation, disinhibition, and paranoid ideation. A provisional diagnosis of medication-induced delirium with hypomanic features was made. Tramadol was identified as a likely precipitating factor, given its opioid and serotonergic properties and the patient’s increased vulnerability as an older adult.

Management initially focused on withdrawal of potentially offending medications, optimisation of physical health, and symptomatic treatment with antipsychotic and benzodiazepine medications. Due to impaired insight, behavioural disturbance, and risk of self-neglect, the patient required detention under the Mental Health Act for assessment and treatment.

Over the course of admission, features typically associated with delirium improved, including confusion and disorientation. However, the patient continued to demonstrate sustained mood elevation, reduced need for sleep, increased goal-directed activity, pressured speech, and intrusive behaviour, with preserved cognition. These symptoms were non-fluctuating and persistent.

Collateral history obtained during admission revealed a possible family history of bipolar affective disorder. In light of the clinical course and longitudinal observation, the diagnosis was revised to a manic episode, likely precipitated by medication exposure and sleep deprivation.

**Results:**

This case illustrates the diagnostic complexity of acute mental state changes in older adults, particularly in the context of polypharmacy and pre-existing affective disorders. While delirium is a common and appropriate initial diagnosis in such presentations, persistence of non-fluctuating manic symptoms following resolution of cognitive disturbance should prompt reconsideration of the diagnosis.

Tramadol has recognised serotonergic and noradrenergic properties and has been associated with both delirium and affective destabilisation, particularly in vulnerable populations. Sleep deprivation may have further contributed to the precipitation of mania. The presence of preserved cognition, sustained mood elevation, and increased goal-directed activity distinguished the evolving presentation from delirium and supported the diagnosis of late-life mania.

This case highlights the importance of longitudinal assessment, collateral history, and diagnostic flexibility when managing behavioural disturbance in older adults.

**Conclusion:**

This case underscores the need to consider late-life mania in the differential diagnosis of acute behavioural disturbance in older adults, particularly when symptoms persist beyond the resolution of delirium. Medication exposure, sleep disruption, and affective vulnerability are important contributing factors. Careful longitudinal observation and willingness to revise initial diagnoses are essential to ensuring accurate diagnosis and appropriate treatment in this population.

